# Preoperative chemotherapy for non-small-cell lung cancer: a systematic review and meta-analysis of individual participant data

**DOI:** 10.1016/S0140-6736(13)62159-5

**Published:** 2014-05-03

**Authors:** NSCLC Meta-analysis Collaborative Group

## Abstract

**Background:**

Individual participant data meta-analyses of postoperative chemotherapy have shown improved survival for patients with non-small-cell lung cancer (NSCLC). We aimed to do a systematic review and individual participant data meta-analysis to establish the effect of preoperative chemotherapy for patients with resectable NSCLC.

**Methods:**

We systematically searched for trials that started after January, 1965. Updated individual participant data were centrally collected, checked, and analysed. Results from individual randomised controlled trials (both published and unpublished) were combined using a two-stage fixed-effect model. Our primary outcome, overall survival, was defined as the time from randomisation until death (any cause), with living patients censored on the date of last follow-up. Secondary outcomes were recurrence-free survival, time to locoregional and distant recurrence, cause-specific survival, complete and overall resection rates, and postoperative mortality. Prespecified analyses explored any variation in effect by trial and patient characteristics. All analyses were by intention to treat.

**Findings:**

Analyses of 15 randomised controlled trials (2385 patients) showed a significant benefit of preoperative chemotherapy on survival (hazard ratio [HR] 0·87, 95% CI 0·78–0·96, p=0·007), a 13% reduction in the relative risk of death (no evidence of a difference between trials; p=0·18, *I*^2^=25%). This finding represents an absolute survival improvement of 5% at 5 years, from 40% to 45%. There was no clear evidence of a difference in the effect on survival by chemotherapy regimen or scheduling, number of drugs, platinum agent used, or whether postoperative radiotherapy was given. There was no clear evidence that particular types of patient defined by age, sex, performance status, histology, or clinical stage benefited more or less from preoperative chemotherapy. Recurrence-free survival (HR 0·85, 95% CI 0·76–0·94, p=0·002) and time to distant recurrence (0·69, 0·58–0·82, p<0·0001) results were both significantly in favour of preoperative chemotherapy although most patients included were stage IB–IIIA. Results for time to locoregional recurrence (0·88, 0·73–1·07, p=0·20), although in favour of preoperative chemotherapy, were not statistically significant.

**Interpretation:**

Findings, which are based on 92% of all patients who were randomised, and mainly stage IB–IIIA, show preoperative chemotherapy significantly improves overall survival, time to distant recurrence, and recurrence-free survival in resectable NSCLC. The findings suggest this is a valid treatment option for most of these patients. Toxic effects could not be assessed.

**Funding:**

Medical Research Council UK.

## Introduction

Worldwide, roughly 1·5 million new cases of lung cancer are diagnosed annually^1^ with about 85% being non-small-cell lung cancers (NSCLCs).^2^ Surgery is thought the best treatment option, but only about 20–25% of tumours are suitable for potentially curative resection.^3^ Two individual participant data meta-analyses^4^ showed that postoperative chemotherapy, with or without radiotherapy, improved survival.

Preoperative chemotherapy has the potential to reduce tumour size, increase operability, and eradicate micrometastases. Chemotherapy might also be more effective when the blood supply to the tumour is still intact before surgical resection, and chemotherapy might be better tolerated if patients are not recovering from major surgery. However, preoperative chemotherapy will delay surgery, and if ineffective, tumours can become unresectable.

The findings of several reviews, based on aggregate data from randomised controlled trials,^5^, ^6^, ^7^, ^8^, ^9^ have suggested preoperative chemotherapy improves survival. However, these reviews all included different combinations of trials, some of which were confounded by the use of chemotherapy in both arms or radiotherapy in one arm, making the specific effects of preoperative chemotherapy difficult to discern. Furthermore, analyses of other outcomes and how effects vary by patient characteristics were not possible with the aggregate data. Therefore, we did a systematic review and meta-analysis of individual participant data to provide more reliable and up-to-date evidence on the effect of preoperative chemotherapy on survival and other key outcomes and whether this varies by patient subgroup.

## Methods

### Design and study selection

Methods were prespecified in a protocol (available on request). Randomised trials comparing chemotherapy with subsequent surgery versus surgery alone were eligible if they started after Jan 1, 1965, and aimed to include chemotherapy-naive NSCLC patients, suitable for surgery, without any previous malignancy. Trials that planned to use postoperative radiotherapy in both arms, or postoperative chemotherapy in the preoperative arm only, were also eligible.

Published and unpublished trials were sought, with no language restrictions, using randomised trial search filters for Medline and Embase^10^ with additional terms for NSCLC and chemotherapy. These searches were supplemented by searching trial registers, conference proceedings, review articles, and reference lists of trial publications (appendix). Collaborators were asked if they knew of any additional trials. Searches were regularly updated until May, 2013.

### Data collection

For all eligible trials and all patients who were randomised, data were sought on the date of randomisation, treatment allocation, type of chemotherapy and number of cycles, age, sex, histology, performance status, date of surgery, extent of resection, clinical and pathological tumour stage, clinical and pathological response, recurrence, survival, cause of death, and date of last follow-up. Standard methods were used to identify missing data and to assess data validity and consistency.^11^ Patterns of treatment allocation and the balance of baseline characteristics by treatment group were used to check randomisation integrity and follow-up of surviving patients was checked to ensure it was up to date and balanced by arm and fed into a risk of bias assessment for each trial.^12^ Any inconsistencies were resolved and the final dataset verified by the relevant trial contact.

### Definition of outcomes

Our primary outcome, overall survival, was defined as the time from randomisation until death (any cause), with living patients censored on the date of last follow-up. Secondary outcomes were recurrence-free survival, time to locoregional and distant recurrence, cause-specific survival, complete and overall resection rates, and postoperative mortality. There were concerns that for patients receiving their surgery immediately in the surgery-alone arm, any recurrences could be identified sooner than in the preoperative chemotherapy arm. This might erroneously suggest a benefit of chemotherapy. Thus, analyses of recurrence outcomes were calculated from a landmark time of 6 months from the date of randomisation to allow for all patients to have completed their allocated treatment.^13^ Events arising within 6 months of randomisation were regarded as events at this landmark time. Recurrence-free survival was defined as time from the landmark date until locoregional recurrence, distant recurrence, or death, whichever happened first. Patients alive without recurrence were censored on the date of last follow-up. To avoid bias from under-reporting of subsequent events, time to locoregional (distant) recurrence was defined as time from the landmark date to first locoregional (distant) recurrence, and patients experiencing previous distant (local) recurrences were censored on the date of distant (local) recurrence. Patients experiencing a locoregional and distant recurrence on the same date were counted in both analyses. For trials that only recorded the first recurrence, patients having a local (distant) recurrence were censored in the analysis of distant (local) recurrence; all other patients without recurrence were censored on the date of death or last follow-up.

We used data on cause of death to assess the effects of chemotherapy on lung and non-lung cancer survival. However, although eight trials supplied these data, only two provided sufficiently detailed information to discriminate between treatment-related and other non-cancer causes, making it impossible to define these outcomes accurately.

The overall resection rate was defined as the proportion of patients having either a complete or incomplete resection. The complete resection rate was defined as the proportion of patients having a complete resection. Postoperative mortality was defined as the proportion of patients dying within 30 days of surgery, and early mortality was defined as death within 6 months of date of randomisation, to allow for completion of all treatment in each arm.

### Statistical analysis

Unless otherwise stated, all analyses were prespecified in the protocol, and done on an intention-to-treat basis. For time-to-event outcomes, we used the log-rank expected number of events and variance to calculate hazard ratio (HR) estimates of effect for each individual trial, which were then combined across trials using a stratified-by-trial, two-stage, fixed-effect model.^14^ The random-effects model^15^ was used to assess the robustness of the results. χ^2^ heterogeneity tests were used to assess differences in the effect of treatment or treatment by covariate interactions across trials. Results for time-to-event outcomes are also presented as non-stratified Kaplan-Meier curves.^16^ The median follow-up was computed for all patients using the reverse Kaplan-Meier method.^17^ For dichotomous outcomes, such as resection rate, the numbers of events and patients were used to calculate Peto odds ratio (OR) estimates of effect^14^ for trials, which were then pooled across trials, using a fixed-effect model.

To explore any effect of trial-level characteristics on the effect of chemotherapy, pooled HRs were calculated for each prespecified trial group. χ^2^ tests for interaction and the *F* ratio were used to assess differences in treatment effect across trial groups. To investigate the effect of patient characteristics on the effect of chemotherapy, the relevant treatment by patient covariate interaction term was included in a Cox regression for each trial. The resulting within-trial interactions (HRs) were then pooled across trials using the stratified-by-trial, fixed-effect model.^18^ These analyses are focused on the primary outcome of survival.

Absolute differences in outcome at 5 years were calculated from the HR and the control group baseline event rate.^19^ All p values are two-sided.

### Role of the funding source

The sponsors of the study had no role in study design, data collection, data analysis, data interpretation, or writing of the report. The corresponding author had full access to all the data in the study and had final responsibility for the decision to submit for publication.

## Results

We identified 19 eligible randomised controlled trials; 17 published^20^, ^21^, ^22^, ^23^, ^24^, ^25^, ^26^, ^27^, ^28^, ^29^, ^30^, ^31^, ^32^, ^33^, ^34^, ^35^, ^36^ and two unpublished 37, 38 (appendix). Data could not be supplied for three trials,^34^, ^35^, ^36^ and one trial only recruited two patients.^37^ Although data were obtained for all 24 patients excluded from the investigators' original analyses, and reinstated in this meta-analysis, data for two other patients could not be obtained. Therefore, this meta-analysis is based on data from 15 trials^20^, ^21^, ^22^, ^23^, ^24^, ^25^, ^26^, ^27^, ^28^, ^29^, ^30^, ^31^, ^32^, ^33^, ^38^ (2385 patients), representing 92% of patients who were randomised, from all known eligible trials. Any risk of bias associated with the randomisation procedure and completeness of outcome data in these 15 trials was judged to be low and the effects of early stopping were minimised by the collection of updated follow-up and investigated in the analyses.

Ten trials^22^, ^24^, ^25^, ^26^, ^27^, ^28^, ^29^, ^30^, ^32^, ^33^ gave chemotherapy only preoperatively and five trials^20^, ^21^, ^23^, ^31^, ^38^ used chemotherapy preoperatively and then postoperatively, usually to responders. All trials used platinum-based chemotherapy, except one,^26^ which used docetaxel alone (table 1). Seven trials^20^, ^21^, ^22^, ^23^, ^24^, ^27^, ^32^ used cisplatin, four^29^, ^30^, ^33^, ^38^ carboplatin, and three^25^, ^28^, ^31^ either cisplatin or carboplatin. Eight trials^21^, ^22^, ^23^, ^24^, ^27^, ^28^, ^30^, ^33^ used postoperative radiotherapy in both arms.

**Table 1:** Trial characteristics

	**Accrual years**	**Number of patients**	**Clinical stage**	**Preoperative chemotherapy used (dose per cycle)**	**Postoperative chemotherapy cycles planned**	**Postoperative radiotherapy planned**	**Reached target accrual**	**Stopping reason**	**Median follow-up (years)**
France 1990^20^	1985–87	26	I–III	Cyclophosphamide (600 mg/m^2^), vindesine (3 mg/m^2^), cisplatin (100 mg/m^2^); 2 cycles every 4 weeks	2	No	No	High progression rate with preoperative chemotherapy	3·2
MD Anderson 1994^21^	1987–93	60	IIIA	Cyclophosphamide (500 mg/m^2^; d1), etoposide (100 mg/m^2^; d1–3), cisplatin (100 mg/m^2^; d1); 3 cycles every 4 weeks	3 to responders	Yes, if surgery incomplete or unresectable	No	Benefit of preoperative chemotherapy	6·7
Spain 1994^22^	1989–91	59	IIIA	Mitomycin (6 mg/m^2^), ifosfamide (3 g/m^2^), cisplatin (50 mg/m^2^); 3 cycles every 3 weeks	0	Yes	No	Benefit of preoperative chemotherapy	6·3
MIP-91^23^	1991–97	355	I–IIIA	Mitomycin (6 mg/m^2^, d1), Ifosfamide (1·5 g/m^2^, d1–3), cisplatin (30 mg/m^2^, d1–3); 2 cycles every 3 weeks	2 to responders	Yes, if surgery incomplete or pT3 or pN2	Yes	NA	12·9
SWOG S9015^38^	1992–94	21	I–IIIA	Etoposide (80 mg/m^2^; d1–3), carboplatin (350 mg/m^2^; d1); 2 cycles every 3 weeks	3 to responders	No	No	Poor accrual	6·3
JCOG 9209^24^	1993–98	62	IIIA	Vindesine (3 mg/m^2^; d1,8), cisplatin (80 mg/m^2^; d1); 3 cycles every 4 weeks	0	Yes, if surgery incomplete	No	Poor accrual	5·7
Netherlands 2000^25^	1994–99	79	IB–II	Paclitaxel (175 mg/m^2^; d1), carboplatin (AUC=7; d1); or teniposide (120 mg/m^2^; d1–3), cisplatin (80 mg/m^2^; d1); at least 2 cycles every 3 weeks	0	No	No	Poor accrual	2·2
Finland 2003^26^	1995–99	62	III	Docetaxel (100 mg/m^2^; d1); 3 cycles every 3 weeks	0	No	No	Poor accrual	3·1
MRC BLT^27^	1995–2001	10	I–III	Vindesine (3 mg/m^2^; d1,8), cisplatin (80 mg/m^2^; d1); or vinorelbine (30 mg/m^2^; d1,8), cisplatin (80 mg/m^2^; d1); or mitomycin (6 mg/m^2^; d1), ifosfamide (3 g/m^2^; d1), cisplatin (50 mg/m^2^; d1); or mitomycin (6 mg/m^2^; d1), vinblastine (6 mg/m^2^; d1), cisplatin (50 mg/m^2^; d1); number of cycles/interval unknown	0	Yes	No	Poor accrual	3·9
MRC LU22^28^	1997–2005	519	I–III	Mitomycin (8 mg/m^2^; first 2 cycles only), vinblastine (6 mg/m^2^; max 10 mg), cisplatin (50 mg/m^2^); or mitomycin (8 mg/m^2^; first 2 cycles only), ifosfamide (3 g/m^2^), cisplatin (50 mg/m^2^); or vinorelbine (30 mg/m^2^; d1,8; max 60 mg), cisplatin (80 mg/m^2^; d1); or paclitaxel (175 mg/m^2^), carboplatin (AUC=5); or gemcitabine (1250 mg/m^2^; d1,8), cisplatin (80 mg/m^2^; d1); or docetaxel (75 mg/m^2^), carboplatin (AUC=6); 3 cycles every 3 weeks	0	Yes, if surgery incomplete or progression	Yes	NA	7·6
SWOG S9900^29^	1999–2004	354	IB–IIIA	Paclitaxel (225 mg/m^2^), carboplatin (AUC=6); 3 cycles every 3 weeks	0	No	No	Positive results of adjuvant chemotherapy trials	5·5
China 2002^30^	1999–2004	55	IIIA	Docetaxel (75 mg/m^2^; d1), carboplatin (AUC=5; d1); 2 cycles every 3 weeks	0	Yes, if surgery incomplete	No	Positive results of adjuvant chemotherapy trials/poor accrual	7·8
China 2005^31^	1999–2004	40	IIIA	Gemcitabine (1200–1250 mg/m^2^; d1,8), cisplatin (30 mg/m^2^; d1–3); or gemcitabine (1200–1250 mg/m^2^; d1,8), carboplatin (AUC=5; d1); 2 cycles every 3 weeks	2 to responders	No	No	Poor accrual	3·3
ChEST^32^	2000–04	270	IB–IIIA	Gemcitabine (1250 mg/m^2^; d1,8), cisplatin (75 mg/m^2^; d1); 3 cycles every 3 weeks	0	No	No	Positive results of adjuvant chemotherapy trials	3·10
NATCH^33^	2000–07	413	IA-IIIA	Paclitaxel (200 mg/m^2^), carboplatin (AUC=6); 3 cycles every 3 weeks	0	Yes, if pathological pN2	Yes	NA	4·8

NA=not applicable. AUC=area under the curve.

Data on age, sex, histology, and stage were provided for all but one trial,^20^ and performance status for 11 trials (table 2).^21^, ^23^, ^25^, ^26^, ^27^, ^28^, ^29^, ^30^, ^32^, ^33^, ^38^ Based on the available data, patients were mostly men (80%) with a median age of 62 years (IQR 55–68) and good performance status (88%). They had mainly clinical stage IB–IIIA tumours (93%) that were predominantly squamous cell carcinomas (50%) or adenocarcinomas (29%). The median follow-up of all patients was 6 years (IQR 4·2–8·2; table 1).

**Table 2:** Characteristics of included patients

	**Surgery**	**Chemotherapy plus surgery**
**Age, years**		
<60	450 (38%)	486 (42%)
60–64	239 (20%)	202 (17%)
65–69	259 (22%)	251 (22%)
≥70	244 (20%)	224 (19%)
Unknown	2 (<1%)	2 (<1%)
**Sex**		
Male	970 (81%)	918 (79%)
Female	221 (19%)	244 (21%)
Unknown	3 (<1%)	3 (<1%)
**Histology**		
Adenocarcinoma	353 (29%)	327 (28%)
Squamous	616 (52%)	573 (49%)
Large cell	49 (4%)	78 (7%)
Other	162 (14%)	176 (15%)
Unknown	14 (1%)	11 (1%)
**Clinical stage**		
IA	63 (5%)	71 (6%)
IB	545 (46%)	501 (43%)
IIA	21 (2%)	29 (3%)
IIB	309 (26%)	278 (24%)
IIIA	246 (21%)	270 (24%)
IIIB	4 (<1%)	9 (<1%)
IV	0 (<1%)	3 (<1%)
Unknown	6 (<1%)	4 (<1%)
**Performance status**		
0	471 (43%)	463 (43%)
1	514 (46%)	494 (45%)
2+	123 (11%)	125 (12%)
Unknown	4 (<1%)	4 (<1%)

Data are n (%). Data for all characteristics, except performance status, were available for 14 of the 15 trials (99% of all patients). For performance status, data were available for 11 of the 15 trials (92% of all patients).

**Figure 1:**
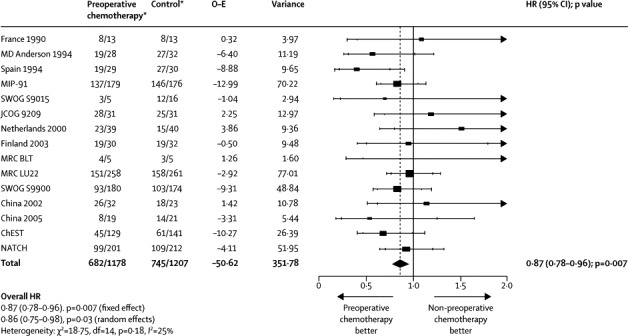
Effect of preoperative chemotherapy on survival Each square denotes the HR for that trial comparison with the horizontal lines showing the 95% and 99% CIs. The size of the square is directly proportional to the amount of information contributed by the trial. The black diamond gives the pooled HR from the fixed effect model; the centre of this diamond denotes the HR and the extremities the 95% CI. O–E=observed minus expected. HR=hazard ratio. MIP=mitomycin, ifosphamide, cisplatin. SWOG=South West Oncology Group. JCOG=Japanese Cancer Oncology Group. MRC=Medical Research Council. BLT=Big Lung Trial. ChEST=Chemotherapy for Early Stages Trial. NATCH=Neoadjuvant/Adjuvant Trial of Chemotherapy. df=degrees of freedom. *Number of events/number entered.

**Figure 2:**
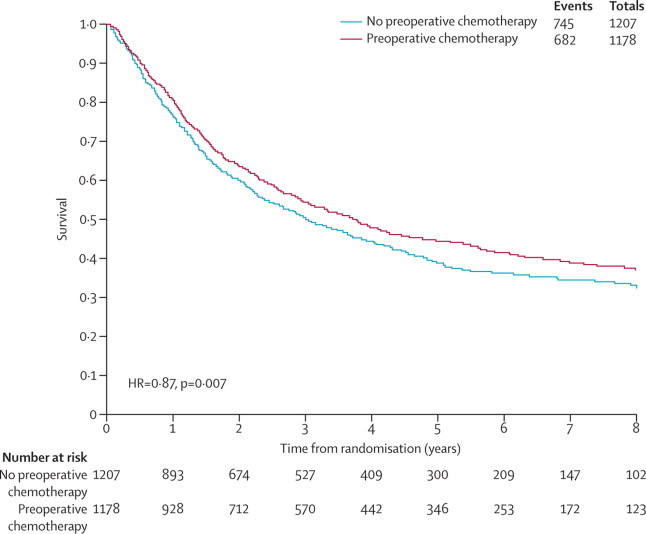
Kaplan-Meier curves (non-stratified) of the effect of preoperative chemotherapy on time to survival

Survival results were based on 15 randomised controlled trials (2385 patients, 1427 deaths) and show a clear benefit of preoperative chemotherapy (HR 0·87, 95% CI 0·78–0·96; p=0·007; Figure 1, Figure 2). This represents a 13% reduction in the relative risk of death, translating to a 5% absolute improvement in survival at 5 years (from 40% to 45%). Despite design differences between trials, for example, a variety of chemotherapy regimens, exclusive use of preoperative chemotherapy, use of postoperative radiotherapy in both arms, and inclusion of all stages of patients or only a specific stage of patient, there was no clear evidence of statistical heterogeneity (p=0·18).

There is no clear evidence that the effect of chemotherapy on survival differed according to whether chemotherapy was given preoperatively or both preoperatively and postoperatively (interaction p=0·23), the number of preoperative chemotherapy cycles (interaction p=0·68), the type of chemotherapy regimen (interaction p=0·94), the number of chemotherapy agents per regimen (interaction p=0·84), or both the type of chemotherapy regimen and number of agents (interaction p=0·79; table 3). Analyses of the type of regimen, the number of agents per regimen, and both the type of regimen and number of agents were repeated only in those trials that gave platinum-based regimens, and gave similar results (interactions p=0·91, p=0·60, and p=0·62 respectively; table 3). We did not identify evidence of a difference in effect of chemotherapy on survival by whether regimens were cisplatin or carboplatin-based (interaction p=0·48) or whether postoperative radiotherapy was used (interaction p=0·87; table 3).

**Table 3:** Effect of preoperative chemotherapy by prespecified trial group

		**Number of trials**	**Number of deaths/patients**	**Hazard ratio (95%CI), p value**	**Heterogeneity p value**	**F ratio p value**	**Interaction p value**
Survival by planned chemotherapy schedule (n=15 trials)						0·32	0·23
	Preoperative chemotherapy only	10	1045/1883	0·90 (0·80–1·02), 0·09	0·10		
	Preoperative and postoperative chemotherapy (to responders)	5	382/502	0·78 (0·64–0·95), 0·02	0·62		
Survival by number of preoperative chemotherapy cycles (n=14 trials)						0·74	0·68
	2 cycles	6	418/576	0·89 (0·74–1·08), 0·25	0·39		
	3 cycles	8	1002/1799	0·85 (0·75–0·96), 0·01	0·10		
Survival by chemotherapy regimen (n=14 trials)						0·96 (all trials), 0·94 (platinum-only trials)	0·95 (all trials), 0·91 (platinum-only trials)
	Platinum plus second generation chemotherapy	7	543/694	0·86 (0·72–1·02), 0·08	0·03		
	Platinum plus third generation chemotherapy	6	801/1540	0·85 (0·74–0·97), 0·02	0·57		
	Non-platinum chemotherapy	1	38/62	0·95 (0·50–1·79), 0·87	NA		
Survival by the number of chemotherapy agents (n=15 trials)						0·90 (all trials), 0·70 (platinum-only trials)	0·84 (all trials), 0·60 (platinum-only trials)
	Non platinum single agent regimen	1	38/62	0·95 (0·50–1·79), 0·87	NA		
	Doublet regimen	9	907/1702	0·88 (0·78–1·01), 0·06	0·42		
	Triplet regimen	5	475/611	Fixed effect 0·83 (0·69–1·00), 0·05; random effects 0·79 (0·53–1·18), 0·25	0·01		
Survival by chemotherapy regimen and number of chemotherapy agents (n=14 trials)						0·89 (all trials), 0·95 (platinum-only trials)	0·79 (all trials), 0·62 (platinum-only trials)
	Non-platinum single agent regimen	1	38/62	0·95 (0·50–1·79), 0·87	NA		
	Platinum second generation, doublet	2	68/83	1·08 (0·66–1·76), 0·76	0·42		
	Platinum second generation, triplet	5	475/611	Fixed effect 0·83 (0·69–1·00), 0·05; random effects 0·79 (0·53–1·18), 0·25	0·01		
	Platinum third generation, doublet	6	801/1540	0·85 (0·74–0·97), 0·02	0·57		
Survival by cisplatin or carboplatin regimen (n=12 trials)						0·54	0·48
	Cisplatin-based	7	830/1289	0·83 (0·72–0·95), 0·01	0·08		
	Carboplatin-based	5	492/905	0·90 (0·75–1·07), 0·23	0·88		
Survival by planned postoperative radiotherapy (n=15 trials)						0·64	0·57
	No postoperative radiotherapy given	8	431/852	0·83 (0·68–1·00), 0·05	0·40		
	Postoperative radiotherapy given	7	996/1533	0·88 (0·78–1·00), 0·05	0·09		
Survival by whether trial stopped early (all trials; n=15 trials)						0·10	0·05
	Reached target accrual	3	800/1287	0·90 (0·79–1·04), 0·16	0·66		
	Stopped for benefit of chemotherapy	2	92/119	0·48 (0·31–0·74), <0·001	0·43		
	Stopped for high progression on chemotherapy arm	1	16/26	1·08 (0·41–2·90), 0·87	NA		
	Stopped for poor accrual/positive adjuvant trials	9	519/953	0·88 (0·74–1·05), 0·17	0·31		

NA=not applicable.

Although the interaction test is not significant there is some suggestion of a larger relative effect in trials where postoperative chemotherapy is given to responders (HR 0·78, 95% CI 0·64–0·95, p=0·02) than in those giving preoperative chemotherapy alone. Exploratory analyses examining whether such an approach modifies the effect of chemotherapy on time to local recurrence showed a similar pattern (preoperative chemotherapy HR 0·94, 95% CI 0·75–1·18, p=0·60; preoperative plus postoperative chemotherapy HR 0·73, 95% CI 0·50–1·07, p=0·11), but again no clear evidence of an interaction (p=0·26). However, for time to distant recurrence, there is evidence of a difference in effect by chemotherapy scheduling (p=0·05), with a substantially greater relative benefit in trials giving postoperative chemotherapy (HR 0·53, 95% CI 0·39–0·73, p<0·001) than in those using just preoperative chemotherapy (HR 0·78, 95% CI 0·63–0·96, p=0·02).

12 trials did not reach their target accrual. Two^21^, ^22^ closed early after recording a benefit of chemotherapy, one^20^ due to high progression rates in the chemotherapy arm, six due to poor accrual^24^, ^25^, ^26^, ^27^, ^31^, ^38^ and three due to positive results in postoperative chemotherapy trials.^29^, ^30^, ^32^ Based on all trials, although we found some evidence of a difference in effect by the reason for early stopping of trials, small trials with extreme positive and negative estimates seem to strongly affect this result (table 3). An exploratory analysis, excluding smaller trials (100 patients or fewer), was based on 80% of the data (77% of all deaths),^23^, ^28^, ^29^, ^32^, ^33^ and showed no clear difference in effect between trials stopping early and those reaching their target accrual (interaction p=0·24).

**Figure 3:**
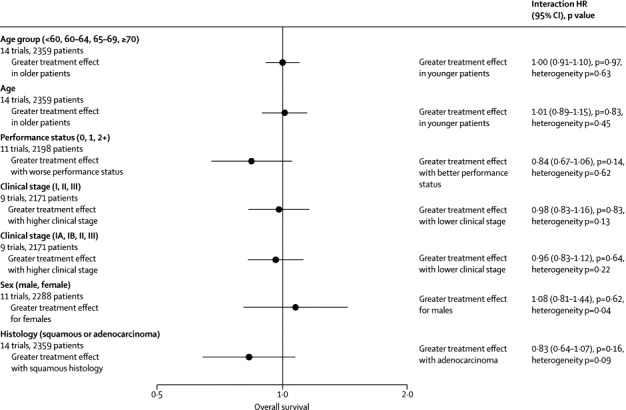
Forest plot of the interactions between the effect of preoperative chemotherapy on survival and covariates The circles represent (fixed effect) meta-analyses of the HRs representing the interactions between the effect of chemotherapy and patient characteristics; the horizontal line shows the 95% CI. HR=hazard ratio.

We did not identify clear evidence that the effect of preoperative chemotherapy on survival differed by age, age group, performance status, or histology (figure 3). Although, overall, there is no evidence of a difference in effect by sex, there is heterogeneity in the interaction (figure 3). Some trials suggest the effect might be greater in women and others in men, but it is not clear why. Also, there was a significant interaction between the effect of preoperative chemotherapy and stage in the ChEST trial,^32^ but not in the other trials, or across all trials (interaction p=0·83; appendix). An exploratory analysis, splitting clinical stage I disease into IA and IB, also identified an interaction between the treatment effect and clinical stage in the ChEST trial, but not across trials (p=0·64, heterogeneity p=0·22). Thus, the overall HR of 0·87 was applied to the control group survival for each stage, giving an absolute survival improvement at 5 years of 5% for all stages, taking it from 50% to 55% in stage I, from 30% to 35% in stage II, and from 20% to 25% in stage III. However, most patients in stage I are IB (89%), in stage II are IIB (92%), and in stage III are IIIA (98%), therefore we can be most confident of results for these patients.

Mortality within 30 days of surgery could be calculated for nine trials,^23^, ^25^, ^26^, ^28^, ^29^, ^30^, ^31^, ^32^, ^38^ (1611 patients, 52 deaths) that supplied date of surgery. Four of these^26^, ^30^, ^31^, ^38^ had no deaths within 30 days of surgery in either arm and an OR was not estimable. Overall, we did not identify a difference between treatment arms (OR 1·48, 95% CI 0·85–2·58, p=0·17; heterogeneity p=0·45, appendix). Based on all 15 trials (2381 patients, 254 deaths), we also did not identify a deleterious effect of preoperative chemotherapy on mortality within 6 months of randomisation (OR 0·88, 95%CI 0·67–1·14, p=0·33; heterogeneity p=0·60).

11 trials^21^, ^23^, ^24^, ^25^, ^26^, ^28^, ^29^, ^30^, ^31^, ^32^, ^38^ (1778 patients) provided data on extent of resection. For the overall resection rate, ORs could not be estimated for four trials^21^, ^23^, ^29^, ^31^ because they had 100% resection rates in both arms. The remaining seven trials^24^, ^25^, ^26^, ^28^, ^30^, ^32^, ^38^ represented less than half of the total data and, with possible variation in the classification of extent of incomplete resection, this analysis was deemed unreliable. Based on all 11 trials, there was no evidence of an effect of preoperative chemotherapy on complete resection (OR 0·88, 95% CI 0·68–1·14, p=0·33; appendix), but the effect did vary between trials (heterogeneity p=0·006). This variation might relate to differences in the types of patients or surgery, because the baseline complete resection rate for control patients ranged from 67% to 95%, with the exception of one trial^21^ where it was substantially lower (31%).

**Figure 4:**
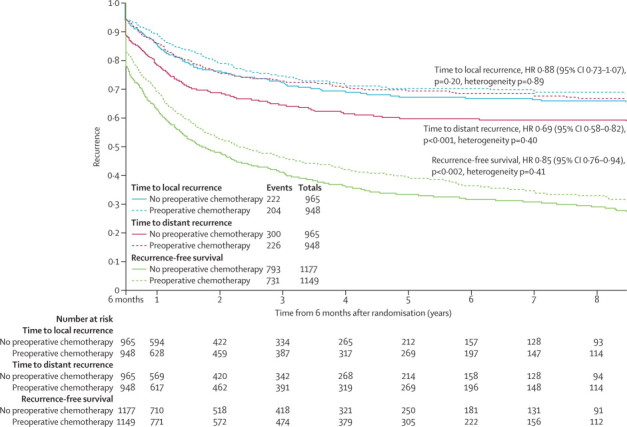
Kaplan-Meier curves (non-stratified) of the effect of preoperative chemotherapy on time to distant and locoregional recurrence and recurrence-free survival Analyses of recurrence outcomes were calculated from a landmark time of 6 months from the date of randomisation; for this reason time on the x-axis starts at 6 months.

Recurrence-free survival data were available for 14 trials^20^, ^21^, ^23^, ^24^, ^25^, ^26^, ^27^, ^28^, ^29^, ^30^, ^31^, ^32^, ^33^, ^38^ (2326 patients, 1524 events). The findings provide clear evidence of a benefit of preoperative chemotherapy (HR 0·85, 95% CI 0·76–0·94, p=0·002, heterogeneity p=0·41, figure 4), translating to an absolute improvement in recurrence-free survival of 6% at 5 years, taking it from 30% to 36%.

Data on both time to locoregional recurrence and distant recurrence were available for 13 trials^20^, ^21^, ^23^, ^24^, ^25^, ^26^, ^27^, ^28^, ^29^, ^30^, ^31^, ^32^, ^38^ and 1913 patients (426 events and 526 events respectively). In these patients, 630 (33%) were alive and free from disease. For the remaining 1283 patients, the first events recorded were locoregional recurrence for 305 (24%), distant recurrence for 397 (31%), both locoregional and distant recurrence for 115 (9%), and death without recurrence for 466 (36%; appendix). There is clear evidence of a benefit of preoperative chemotherapy on time to distant recurrence (HR 0·69, 95% CI 0·58–0·82; p<0·001; heterogeneity p=0·40; figure 4), but the effect on time to locoregional recurrence was less clear (HR 0·88, 95% CI 0·73–1·07; p=0·20; heterogeneity p=0·89; figure 4). These findings translate into an absolute improvement in time to distant recurrence of 10% at 5 years (from 60% to 70%). There is a potential improvement on time to locoregional recurrence of 3% at 5 years.

## Discussion

Based on data from 15 randomised trials (92% of all patients who were randomised), we have shown a 5% absolute benefit of preoperative chemotherapy on 5 year survival in patients with resectable NSCLC. There was no clear evidence of a difference in this effect by treatment type, scheduling, trial design differences, or by patient characteristics, although the results are most reliable for stage IB–IIIA. There seemed to be no excess of early mortality in the preoperative chemotherapy arm as a result of deferred surgery.

Although this meta-analysis included most patients known to have been randomised, four eligible trials (198 patients) could not be included. We could estimate an HR^39^ for survival for one trial of 90 patients,^36^ but not the remaining three trials. Two of these^34^, ^35^ (106 patients) did not report the appropriate information, and one (two patients) was unpublished.^37^ When the single estimated HR was combined with the overall result for the meta-analysis, the effect on survival remained the same (HR 0·87, p=0·006), but being based on 96% of patients who were randomised, it provides more convincing evidence of a benefit of preoperative chemotherapy. This systematic review and meta-analysis will be updated if further eligible trials are identified.

One reason for using preoperative chemotherapy is that it might make tumours more operable, potentially improving the likelihood of a complete resection. Conversely, delays to surgery could make it harder to achieve a complete resection. However, we did not identify clear evidence of a positive or negative effect of chemotherapy on the complete resection rate or a benefit on locoregional recurrence. However, we did note a 10% absolute benefit of preoperative chemotherapy on distant recurrence at 5 years, suggesting that it might have greater potential to eradicate micrometastases than postoperative chemotherapy, where the absolute benefit was 5% at 5 years.^4^

Comparing the effect of preoperative and postoperative chemotherapy directly, using data from this meta-analysis and two previous ones of postoperative chemotherapy in NSCLC proved problematic. Although it was possible to make the datasets comparable in terms of the regimens used, we could not make them comparable in terms of their patient characteristics, particularly stage. Only pathological stage was available for the postoperative chemotherapy meta-analysis, and agreement between clinical and pathological staging in the control group patients of the current meta-analysis was only around 60%. However, survival in the control group of the present meta-analysis is somewhere between that noted for patients receiving surgery alone and those receiving surgery plus radiotherapy as definitive treatment,^4^ suggesting that the present population spans the two. Although this difference makes a formal indirect comparison of the effects of preoperative and postoperative chemotherapy difficult, the benefit noted is on a similar scale. Others have attempted formal comparison based on aggregate data^8^ and concluded the effect of chemotherapy on overall or recurrence-free survival is similar, irrespective of chemotherapy timing. However, they did not include key large trials, published more recently, and have included a trial confounded by the use of radiotherapy in only one arm.^40^

We included one three-arm trial (NATCH^33^) with both preoperative and postoperative chemotherapy arms, but because it was underpowered, the authors did not report their direct comparison. Nevertheless, they provided us with analyses showing similar effects of preoperative and postoperative chemotherapy on survival (HR 0·93, 95% CI 0·71–1·23, p=0·61) and recurrence-free survival (HR 0·88, 95% CI 0·68–1·13, p=0·31; Rosell R, unpublished). Similarly, a recent trial^41^ (198 patients), of preoperative versus postoperative chemotherapy reported no difference in disease-free survival (HR 0·88, 95% CI 0·58–1·33, p=0·54), although power could also be an issue in this trial.

The findings of NATCH^33^ showed a difference in treatment compliance between the preoperative (90%) and the postoperative (60%) chemotherapy arms. Of the trials included in our report, the ten^20^, ^22^, ^23^, ^24^, ^25^, ^26^, ^28^, ^29^, ^32^, ^33^ that reported the number of patients receiving all scheduled preoperative chemotherapy (2–3 cycles), identified a similarly high compliance rate with preoperative chemotherapy (mean compliance rate 85%, range 71–100%). By contrast, for the 14 trials in the postoperative chemotherapy systematic review^4^ that reported patients receiving scheduled chemotherapy (2–6 cycles), the mean compliance rate was somewhat lower (62%, range 41–98%). This implies that patients might receive more of their planned chemotherapy if it is given before surgery.

The results so far seem to suggest similar effects with either preoperative or postoperative chemotherapy, giving a choice of treatment options. Clinicians might consider that preoperative chemotherapy is preferable for poorer prognosis patients with larger, more advanced stage tumours, less able to tolerate chemotherapy after surgery, or in regions where surgery waiting lists are longer. Postoperative chemotherapy might be preferred by surgeons and by patients wishing to have potentially curative treatment immediately, or for those with earlier stage disease. It also allows for more reliable pathological staging to establish if subsequent chemotherapy is appropriate.

Because this meta-analysis shows that preoperative chemotherapy has a greater effect on metastases, and a previous one^4^ shows that postoperative chemotherapy has a greater effect on local control, it is tempting to speculate that combined preoperative and postoperative chemotherapy would confer a greater benefit on local and distant control and survival. This is not entirely borne out by the present survival results by chemotherapy scheduling and generally only those patients responding to preoperative chemotherapy were also given postoperative chemotherapy such that most would have received preoperative chemotherapy alone. However, exploratory analyses do suggest a synergistic effect of combining preoperative and postoperative chemotherapy on time to metastases. However, it should be noted that more cycles of chemotherapy were planned in the trials of combined preoperative and postoperative chemotherapy (2–3 plus 2–3 cycles postoperatively) compared with those of just preoperative chemotherapy (2–3). Moreover, a recently reported trial that compared the use of preoperative chemotherapy plus postoperative chemotherapy^42^ to responders with postoperative chemotherapy in 528 similar patients identified no evidence that preoperative plus postoperative chemotherapy was better (HR 1·01, 95% CI 0·79–1·30, p=0·92). Nevertheless, further head-to-head comparisons of these approaches might be warranted.

The potential benefit of preoperative chemotherapy would need to be balanced against possible toxic effects. However, although we were unable to assess toxic effects at the patient level in this study, trial reports for 13 of the included trials described mild or acceptable toxic effects and that chemotherapy was generally well tolerated. Further questions regarding which drugs to use, the duration of chemotherapy, and if the effect might be modified by predictive genetic biomarkers will need to be answered by new or ongoing trials. Nevertheless, these results provide the most complete evidence so far of the effects of preoperative chemotherapy, showing a significant improvement in overall survival, time-to-distant recurrence, and recurrence-free survival.

Correspondence to: Sarah Burdett, MRC Clinical Trials Unit at UCL, Meta-analysis Group, Aviation House, 125 Kingsway, London WC2B 6NH, UK sarah.burdett@ucl.ac.uk

## References

[1] Jemal A, Bray F, Center MM, Ferlay J, Ward E, Forman D (2011). Global cancer statistics. CA Cancer J Clin.

[2] American Cancer Society (2007). Cancer facts and figures 2007.

[3] Datta D, Lahiri B (2003). Preoperative evaluation of patients undergoing lung resection surgery. Chest.

[4] NSCLC Meta-analyses Collaborative Group (2010). Adjuvant chemotherapy, with or without postoperative radiotherapy, in operable non-small-cell lung cancer: two meta-analyses of individual patient data. Lancet.

[5] Berghmans T, Paesmans M, Meert AP (2005). Survival improvement in resectable non-small cell lung cancer with (neo)adjuvant chemotherapy: results of a meta-analysis of the literature. Lung Cancer.

[6] Nakamura H, Kawasaki N, Taguchi M, Kabasawa K (2006). Role of preoperative chemotherapy for non-small-cell lung cancer: a meta-analysis. Lung Cancer.

[7] Burdett S, Stewart LA, Rydzewska L (2006). A systematic review and meta-analysis of the literature: chemotherapy and surgery versus surgery alone in non-small cell lung cancer. J Thorac Oncol.

[8] Lim E, Harris G, Patel A, Adachi I, Edmonds L, Song F (2009). Preoperative versus postoperative chemotherapy in patients with resectable non-small cell lung cancer: systematic review and indirect comparison meta-analysis of randomized trials. J Thorac Oncol.

[9] Song WA, Zhou NK, Wang W (2010). Survival benefit of neoadjuvant chemotherapy in non-small cell lung cancer: an updated meta-analysis of 13 randomized control trials. J Thorac Oncol.

[10] Lefebvre C, Manheimer E, Glanville J, Higgins JPT, Green S, the Cochrane Information Retrieval Methods Group (2008). Searching for studies. Cochrane Handbook for Systematic Reviews of Interventions.

[11] Stewart LA, Clarke MJ (1995). Practical methodology of meta-analyses (overviews) using updated individual patient data. Cochrane Working Group. Stat Med.

[12] Moher D, Liberati A, Tetzlaff J, Altman DG, the PRISMA Group (2009). Preferred reporting items for systematic reviews and meta-analyses: the PRISMA statement. PLoS Med.

[13] Allum WH, Stenning SP, Bancewicz J, Clark PI, Langley RE (2009). Long-term results of a randomized trial of surgery with or without preoperative chemotherapy in esophageal cancer. J Clin Oncol.

[14] Yusuf S, Peto R, Lewis J, Collins R, Sleight P (1985). Beta blockade during and after myocardial infarction: an overview of the randomized trials. Prog Cardiovasc Dis.

[15] DerSimonian R, Laird N (1986). Meta-analysis in clinical trials. Control Clin Trials.

[16] Kaplan EL, Meier P (1958). Nonparametric estimation from incomplete observation. J Am Stat Assoc.

[17] Schemper M, Smith TL (1996). A note on quantifying follow-up in studies of failure time. Control Clin Trials.

[18] Fisher DJ, Copas AJ, Tierney JF, Parmar MKB (2011). A critical review of methods for the assessment of patient-level interactions in individual participant data meta-analysis of randomized trials, and guidance for practitioners. J Clin Epidemiol.

[19] Stewart LA, Parmar MKB (1993). Meta-analysis of the literature or of individual patient data: is there a difference?. Lancet.

[20] Dautzenberg B, Benichou J, Allard P (1990). Failure of the perioperative PCV neoadjuvant polychemotherapy in resectable bronchogenic non-small cell carcinoma. Results from a randomized phase II trial. Cancer.

[21] Roth JA, Fossella F, Komaki R (1994). A randomized trial comparing perioperative chemotherapy and surgery with surgery alone in resectable stage IIIA non-small-cell lung cancer. J Natl Cancer Inst.

[22] Rosell R, Gómez-Codina J, Camps C (1994). A randomized trial comparing preoperative chemotherapy plus surgery with surgery alone in patients with non-small-cell lung cancer. N Engl J Med.

[23] Depierre A, Milleron B, Moro-Sibilot D, the French Thoracic Cooperative Group (2002). Preoperative chemotherapy followed by surgery compared with primary surgery in resectable stage I (except T1N0), II, and IIIa non-small-cell lung cancer. J Clin Oncol.

[24] Nagai K, Tsuchiya R, Mori T, the Lung Cancer Surgical Study Group of the Japan Clinical Oncology Group (2003). A randomized trial comparing induction chemotherapy followed by surgery with surgery alone for patients with stage IIIA N2 non-small cell lung cancer (JCOG 9209). J Thorac Cardiovasc Surg.

[25] Splinter TA, van Putten JW, Meuzalaar J, Smit EF, Kho GS, Groen HJ (2000). Randomized multicenter phase II study of chemotherapy followed by surgery versus surgery alone in stage I and II non-small cell lung cancer (NSCLC). Proc Am Soc Clin Oncol.

[26] Mattson KV, Abratt RP, ten Velde G, Krofta K (2003). Docetaxel as neoadjuvant therapy for radically treatable stage III non-small-cell lung cancer: a multinational randomised phase III study. Ann Oncol.

[27] Waller D, Peake MD, Stephens RJ (2004). Chemotherapy for patients with non-small cell lung cancer: the surgical setting of the Big Lung Trial. Eur J Cardiothorac Surg.

[28] Gilligan D, Nicolson M, Smith I (2007). Preoperative chemotherapy in patients with resectable non-small cell lung cancer: results of the MRC LU22/NVALT 2/EORTC 08012 multicentre randomised trial and update of systematic review. Lancet.

[29] Pisters KM, Vallières E, Crowley JJ (2010). Surgery with or without preoperative paclitaxel and carboplatin in early-stage non-small-cell lung cancer: Southwest Oncology Group Trial S9900, an intergroup, randomized, phase III trial. J Clin Oncol.

[30] Wu Y-L, Gu L-J, Weng Y-M, Feng W-N, Cheng C (2002). Neo-adjuvant chemotherapy with docetaxel plus carboplatin for non-small cell lung cancer. Ann Oncol.

[31] Yang X, Wu Y, Gu L (2005). A randomized trial comparing neoadjuvant gemcitabine plus carboplatin or cisplatin followed by surgery with surgery alone in Clinical Stage IIIA non-small-cell lung cancer (NSCLC). Lung Cancer.

[32] Scagliotti GV, Pastorino U, Vansteenkiste JF (2012). Randomized phase III study of surgery alone or surgery plus preoperative cisplatin and gemcitabine in stages IB to IIIA non-small-cell lung cancer. J Clin Oncol.

[33] Felip E, Rosell R, Maestre JA, the Spanish Lung Cancer Group (2010). Preoperative chemotherapy plus surgery versus surgery plus adjuvant chemotherapy versus surgery alone in early-stage non-small-cell lung cancer. J Clin Oncol.

[34] de Boer RH, Smith IE, Pastorino U (1999). Pre-operative chemotherapy in early stage resectable non-small-cell lung cancer: a randomized feasibility study justifying a multicentre phase III trial. Br J Cancer.

[35] Yi X, Zhang R, Ding J, Gao W, Ma Q, Zhong C (2003). A clinicopathologic study on neoadjuvant chemotherapy in the treatment of non-small-cell lung cancer. Zhongguo Fei Ai Za Zhi.

[36] Sorensen JB, Riska H, Ravn J (2005). Scandinavian phase III trial of neoadjuvant chemotherapy in NSCLC stages IB-IIIA/T3. Proc Am Soc Clin Oncol.

[37] Chaudhri N Randomized phase III trial of surgery alone or surgery plus preoperative gemcitabine-cisplatin in clinical early stages of non-small cell lung cancer. http://clinicaltrials.gov/show/NCT00540280.

[38] Bunn P Phase III randomized comparison of pre- and postoperative chemotherapy with VP-16/CBDCA vs surgery alone in patients with operable nonsmall cell carcinoma of the lung. http://www.cancer.gov/clinicaltrials/search/view?cdrid=77308&version=HealthProfessional.

[39] Parmar MKB, Torri V, Stewart L (1998). Extracting summary statistics to perform meta-analyses of the published literature for survival endpoints. Stat Med.

[40] Pass HI, Pogrebniak HW, Steinberg SM, Mulshine J, Minna J (1992). Randomized trial of neoadjuvant therapy for lung cancer: interim analysis. Ann Thorac Surg.

[41] Yang X-N, Cheng G, Ben X-S (2013). Survival study of neoadjuvant versus adjuvant chemotherapy with docetaxel combined carboplatin in resectable stage IB to IIIA non-small cell lung cancer. ASCO Annual Meeting.

[42] Westeel V, Quoix E, Puyraveau M, the Intergroupe Francophone de Cancérologie Thoracique (2013). A randomised trial comparing preoperative to perioperative chemotherapy in early-stage non-small-cell lung cancer (IFCT 0002 trial). Eur J Cancer.

