# Supplementary material for: Preoperative chemotherapy for non-small-cell lung cancer: a systematic review and meta-analysis of individual participant data
Source: Lancet. 2014 May 3;383(9928):1561–71. doi: 10.1016/S0140-6736(13)62159-5 (PMC4022989; doi:10.1016/S0140-6736(13)62159-5)
Supplement: Supplementary appendix [file mmc1.pdf]

THE LANCET

Supplementary appendix

This appendix formed part of the original submission and has been peer reviewed. We post it as supplied by the authors.

Supplement to: NSCLC Meta-analysis Collaborative Group. Preoperative chemotherapy for non-small cell lung cancer: a systematic review and meta-analysis of individual participant data. *Lancet* 2014; published online Feb 25. http://dx.doi.org/10.1016/S0140-6736(13)62159-5.

Web Table 1: Effect of pre-operative chemotherapy on mortality and resection rates

Outcome	Number of trials	Number of patients/events	OR (95% CI), p-value	Heterogeneity p-value
All-cause mortality within 30 days of surgery	9	52/1467	1.48 (0.85-2.58), 0.17	0.45
All-cause mortality within 6 months of randomisation	15	254/2381	0.88 (0.67-1.14), 0.33	0.60
Complete resection rate by treatment arm	11	1461/1778	Fixed Effect<br>0.88 (0.68-1.14), 0.33<br>Random Effects<br>0.77 (0.50-1.20), 0.25	0.006

Web Figure 1: PRISMA flow diagram of randomised controlled trial identification, screening, eligibility and inclusion

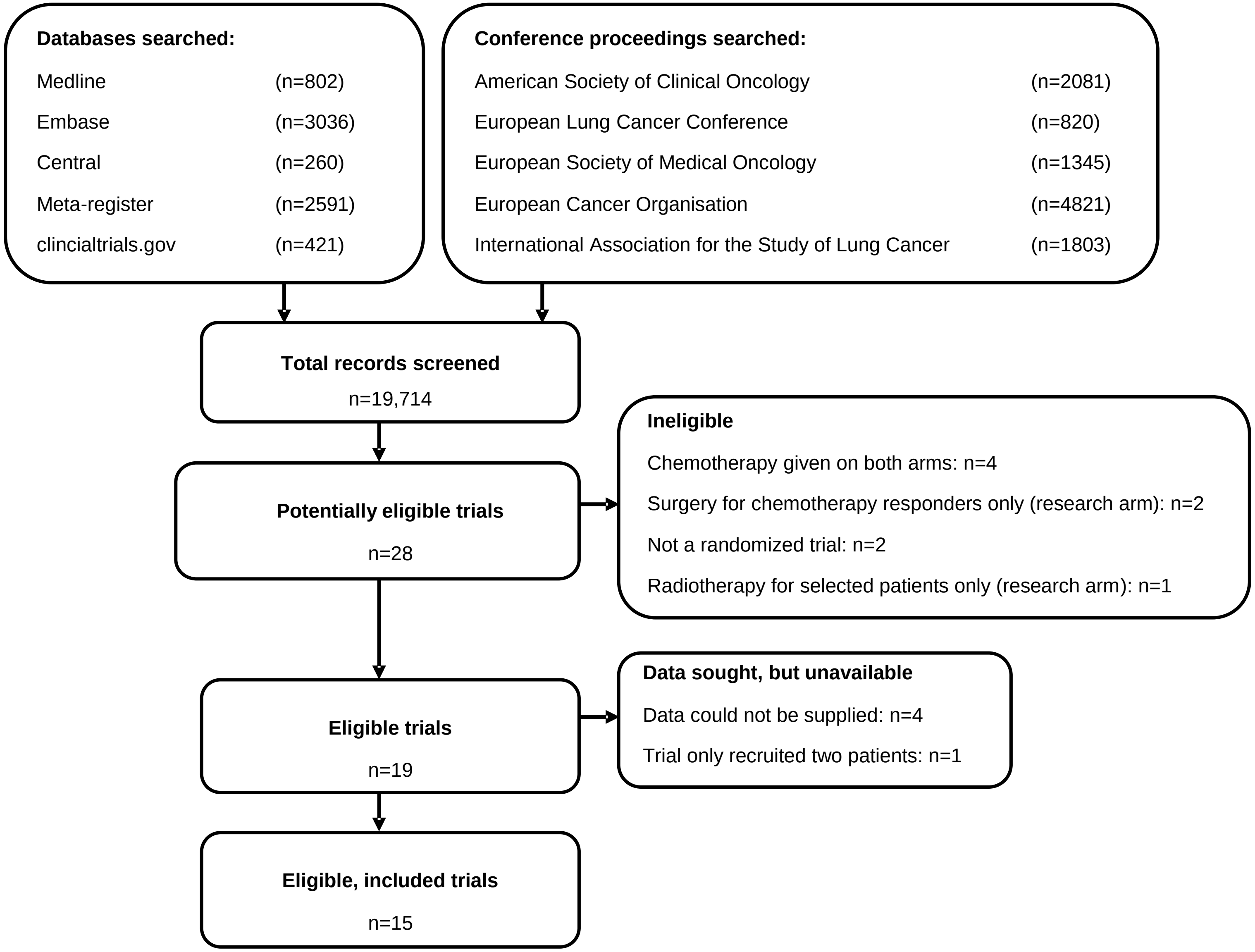

Web Figure 2: Forest plot of the interactions between the effect of pre-operative chemotherapy on survival and stage, by trial and overall

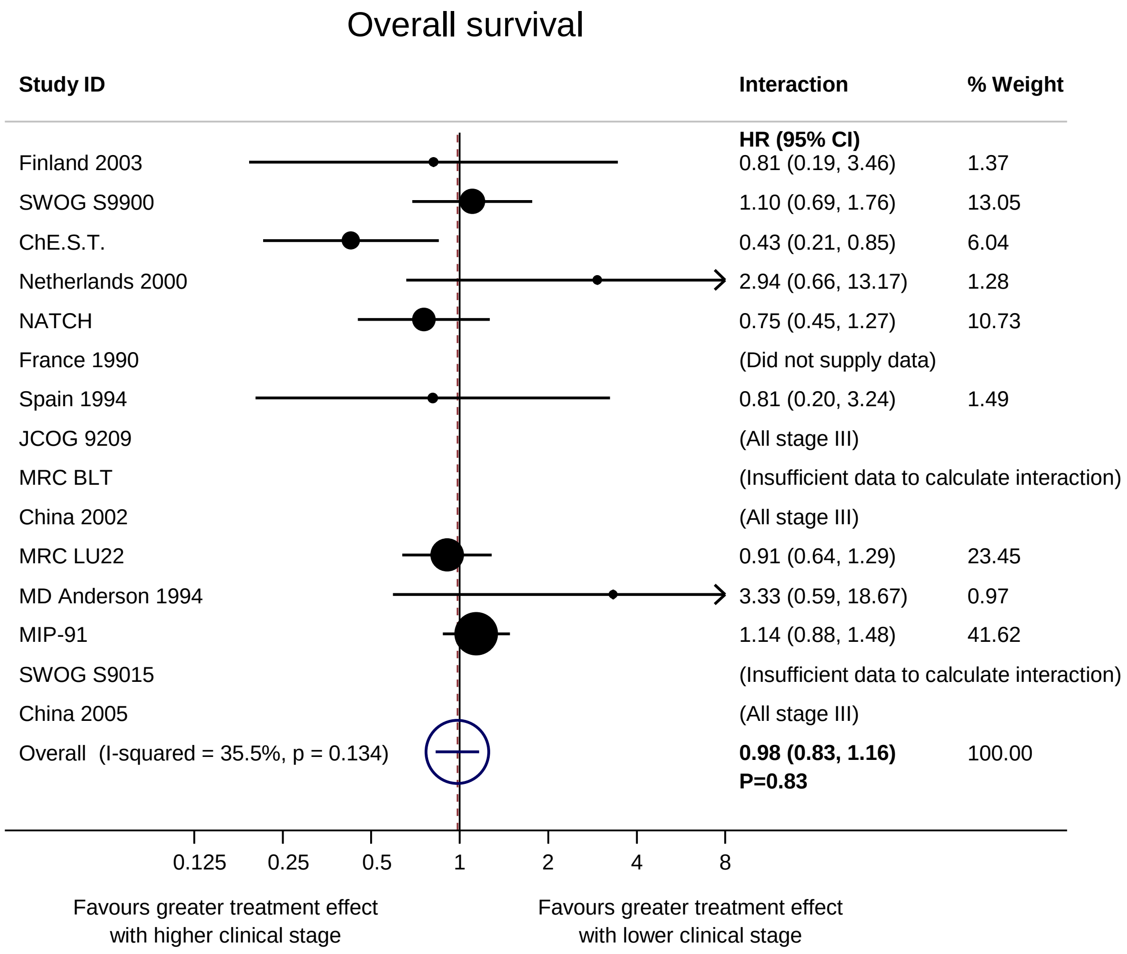

The centre of each filled circle denotes the HR for the interaction between the effect of chemotherapy and stage for each trial, with the horizontal line showing the 95% confidence interval (CI). The size of each circle is directly proportional to the amount of information contributed by a trial. The open circle represents a (fixed effect) meta-analyses of the interaction HRs, again with the horizontal line showing the 95% CI.

Web figure 3: Patterns of loco-regional recurrence, distant recurrence and death

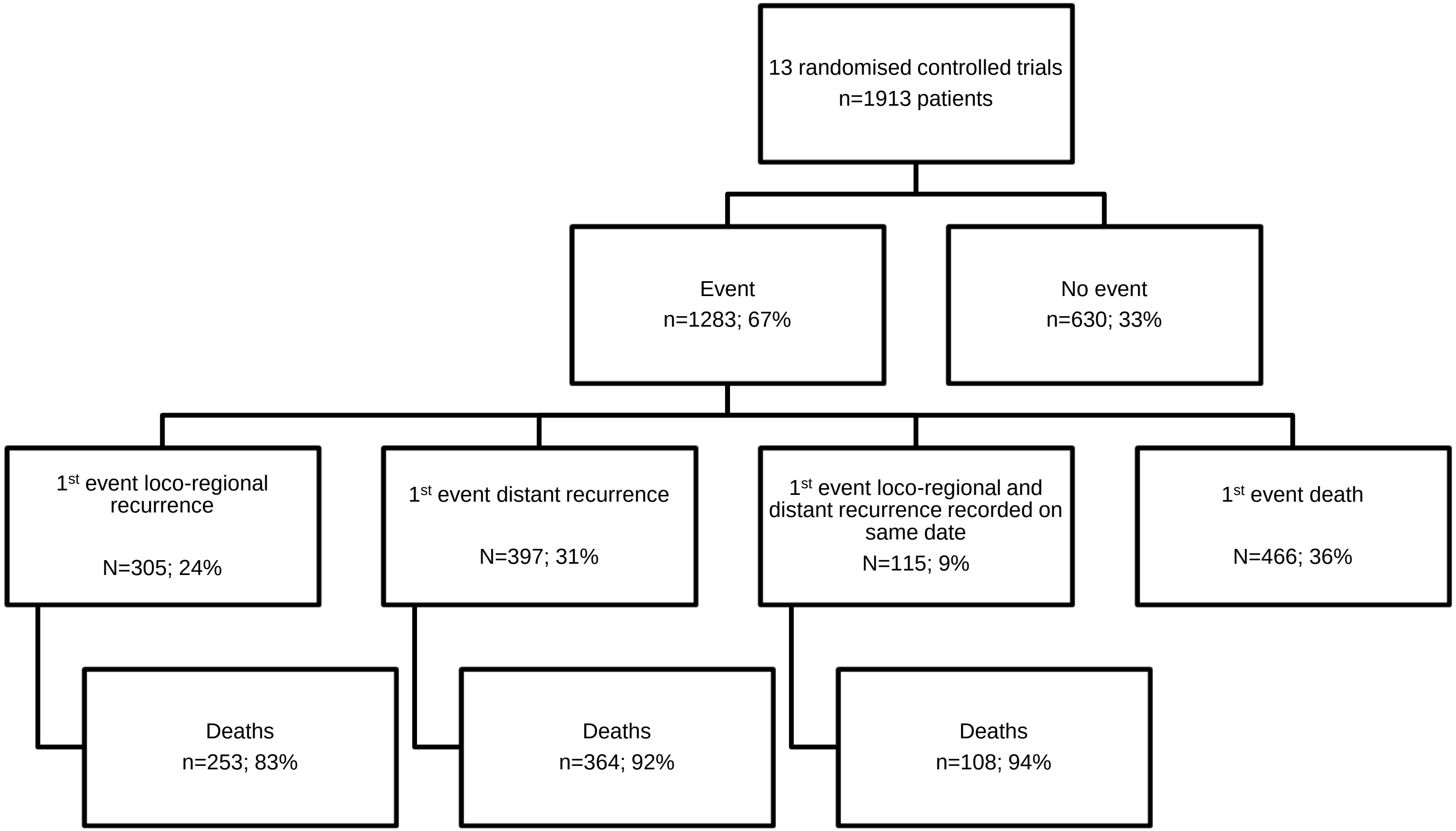

Web Appendix 1: Search strategies

Cochrane Highly Sensitive Search Strategy for identifying randomised controlled trials (MEDLINE)[1]

1. "randomi*ed controlled trial".pt.

2. controlled clinical trial.pt.

3. "randomi*ed".ab.

4. placebo.ab.

5. drug therapy.fs.

6. randomly.ab.

7. trial.ab.

8. groups.ab.

9. 1 or 2 or 3 or 4 or 5 or 6 or 7 or 8

10. (animals not (humans and animals)).sh.

11. 9 not 10

Terms specific to lung cancer:

12. exp Lung Neoplasms/

13. exp Carcinoma, Non-Small-Cell Lung/

14. (lung$ adj3 canc$).mp.

15. (lung$ adj3 carcinoma$).mp.

16. (lung$ adj3 tumo?r$).mp.

17. (lung$ adj3 neoplasm$).mp.

18. 12 or 13 or 14 or 15 or 16 or 17

Terms specific to the intervention:

19. exp Drug Therapy/

20. exp Neoadjuvant.mp

21. chemotherapy.mp.

22. 19 or 20 or 21

23. exp General Surgery/

24. surgery.mp.

25. 23 or 24

Combination of terms to identify randomised controlled trials of chemotherapy and surgery in lung cancer

26. 11 and 18 and 22 and 25

Best optimisation of sensitivity/specificity combination strategy for identifying randomised controlled trials (EMBASE)[2]

1. random:.tw.
2. placebo:.mp.
3. double-blind:.tw.
4. or/1-3

Terms specific to lung cancer:

5. exp lung tumour/
6. lung neoplasms.mp.
7. exp lung non small cell cancer/
8. (lung: adj3 canc:).mp.
9. (lung: adj3 carcinoma:).mp.
10. (lung: adj3 tumo?r:).mp.
11. (lung: adj3 neoplasm:).mp.
12. or/5-11

Terms specific to the intervention:

13. exp drug therapy/
14. neoadjuvant therapy.mp.
15. chemotherapy/
16. chemotherapy.mp.
17. or/13-16
18. exp surgery/
19. surgery.mp.
20. or/18-19

Combination of terms to identify randomised controlled trials of chemotherapy and surgery in lung cancer

21. 4 and 12 and 17 and 20

Search strategy for identifying randomised controlled trials (CENTRAL)

1. MeSH descriptor: [Lung Neoplasms] explode all trees
2. lung near canc*:ti,ab,kw
3. (lung near carcinoma*):ti,ab,kw
4. lung near neoplasm*:ti,ab,kw
5. lung near tumo?r*:ti,ab,kw
6. (non small cell):ti,ab,kw
7. non-small cell:ti,ab,kw
8. #1 or #2 or #3 or #4 or #5 or #6 or #7
9. MeSH descriptor: [Drug Therapy] explode all trees
10. chemotherapy:ti,ab,kw
11. #8 or #9
12. lobectomy:ti,ab,kw
13. pneumonectomy:ti,ab,kw
14. MeSH descriptor: [Thoracic Surgery] explode all trees
15. MeSH descriptor: [General Surgery] explode all trees
16. surgery:ti,ab,kw
17. #12 or #13 or #14 or #15 or #16
18. #8 and #11 and #17
19. #8 and #11 and #17 from 2009 to 2012

The MetaRegister was searched using “(NSCLC or lung cancer) AND chemotherapy” as keywords and the Physicians Data Query (PDQ) and Clinical Trials.gov registers were searched using “lung cancer AND surgery AND chemotherapy” as keywords

References

1 Higgins JPT, Green SJ, eds. Cochrane Handbook for Systematic Reviews of Interventions. Chichester: John Wiley & Sons Ltd; 2008.

2 Wong SS, Wilczynski NL, Haynes RB. Developing optimal search strategies for detecting clinically sound treatment studies in EMBASE. *J Med Libr Assoc* 2006; **94:** 41–47.
